# Quantitative Colorimetric Sensing of Carbidopa in Anti-Parkinson Drugs Based on Selective Reaction with Indole-3-Carbaldehyde

**DOI:** 10.3390/s23229142

**Published:** 2023-11-13

**Authors:** Pasquale Palladino, Alberto Rainetti, Mariagrazia Lettieri, Giuseppe Pieraccini, Simona Scarano, Maria Minunni

**Affiliations:** 1 Department of Chemistry ‘Ugo Schiff’, University of Florence, Via della Lastruccia 3-13, 50019 Sesto Fiorentino, Italy; 2 Department of Biotechnology, Chemistry and Pharmacy, University of Siena, Via Aldo Moro, 2, 53100 Siena, Italy; 3 CISM Mass Spectrometry Centre, University of Florence, Viale Gaetano Pieraccini 6, 50139 Florence, Italy; 4 Department of Pharmacy, University of Pisa, Via Bonanno Pisano, 6, 56126 Pisa, Italy

**Keywords:** indole-3-carbaldehyde, carbidopa, levodopa, aldazine, anti-Parkinson drugs, colorimetry

## Abstract

The quality of life of patients affected by Parkinson’s disease is improved by medications containing levodopa and carbidopa, restoring the dopamine concentration in the brain. Accordingly, the affordable quality control of such pharmaceuticals is very important. Here is reported the simple and inexpensive colorimetric quantification of carbidopa in anti-Parkinson drugs by the selective condensation reaction between the hydrazine group from carbidopa and the formyl functional group of selected aldehydes in acidified hydroalcoholic solution. An optical assay was developed by using indole-3-carbaldehyde (I3A) giving a yellow aldazine in EtOH:H_2_O 1:1 (λ_max_~415 nm) at 70 °C for 4 h, as confirmed by LC-MS analysis. A filter-based plate reader was used for colorimetric data acquisition, providing superior results in terms of analytical performances for I3A, with a sensitivity ~50 L g^−1^ and LOD ~0.1 mg L^−1^ in comparison to a previous study based on vanillin, giving, for the same figures of merit values, about 13 L g^−1^ and 0.2–0.3 mg L^−1^, respectively. The calibration curves for the standard solution and drugs were almost superimposable, therefore excluding interference from the excipients and additives, with very good reproducibility (_av_RSD% 2–4%) within the linear dynamic range (10 mg L^−1^–50 mg L^−1^).

## 1. Introduction

Known since ancient times, Parkinson’s disease (PD) is the most common movement disorder [1], named after the British physician, geologist, and paleontologist James Parkinson, who firstly described in 1817 individuals with pathognomonic symptoms of the progressive and chronic disease, i.e., rest tremors, bradykinesia, rigidity, and loss of postural reflexes, although a more complete and accurate description of the disease was already published in 1690 by Ferenc Pápai Páriz [2]. Recent studies have allowed to shed light on the genetic, epigenetic, and environmental factors that influence the predisposition to the second-most common age-related neurodegenerative disease [3,4,5,6] after Alzheimer’s one, with molecular mechanisms resembling those observed in prion diseases [7,8,9]. PD affects millions of people worldwide, up to 2% over the age of sixty-five, prevalently men from Europe and North America [10,11,12].

**Figure 1:**
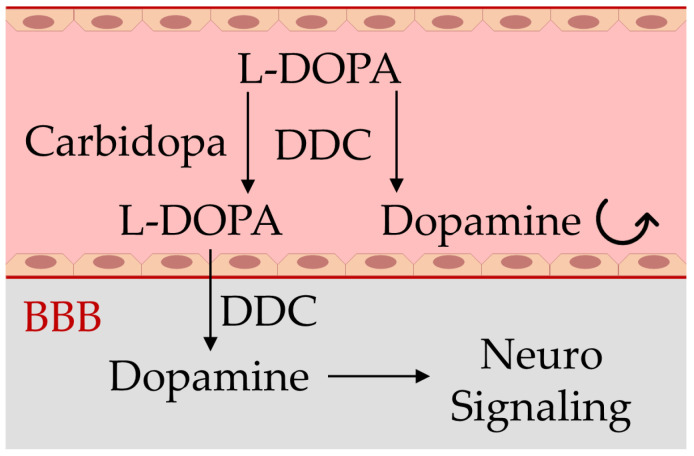
Schematic representation of the blood–brain barrier (BBB) impairing dopamine crossing from blood (light red) to the brain (gray), and the mechanism of action of the drugs used in Parkinson’s disease containing carbidopa and levodopa (L-DOPA). Carbidopa limits the premature transformation of L-DOPA to dopamine in blood by the enzyme dopa-decarboxylase (DDC), allowing a larger dopamine generation in the brain and the subsequent signaling activity restoration of this neurotransmitter [5,16,17].

In PD patients, dopaminergic neurons are damaged, and this leads to a deficiency in dopamine neurotransmitters, impairing the proper body movements and coordination but also damaging the working memory, feeling of pleasure, sleep regulation, and cognitive faculties, compromising the patient’s autonomy and quality of life. Although there is still no cure for PD, several therapeutic strategies are employed in laboratory and clinical settings, including the mitigation of neuroinflammation [13,14,15], and, mainly, drug therapy based on restoration of the dopamine levels within the brain can lead to temporary symptomatic relief of motor symptoms of the disease and increase the life expectancy of patients [10,12]. However, because dopamine is not able to cross the blood–brain barrier (BBB), in place of this neurotransmitter, its precursor levodopa (L-DOPA) represents the active ingredient of some pharmacological formulations for the treatment of PD, together with carbidopa or benserazide, which are peripheral DOPA-decarboxylase inhibitors that increase the L-DOPA availability within dopaminergic neurons, where it is converted to dopamine (Figure 1), requiring lower doses and reducing adverse side effects [5,16,17].

Accordingly, the affordable quality control of such pharmaceutical formulations is very important, and it is currently achieved by means of several methodologies [16,17,18,19,20,21,22,23,24,25,26], including HPLC, obtaining an effective separation and quantification of levodopa, carbidopa, benserazide, and entacapone [16]; capillary zone electrophoresis with UV detection, suitable for the quality control of drugs containing levodopa with carbidopa or benserazide [17]; chemometrics-assisted spectrophotometric methods, developed for the simultaneous quantitative determination of levodopa, carbidopa, and methyldopa [20,21,22]; and voltammetry, giving the electrochemical quantification of levodopa and carbidopa in pharmaceutical products after filtration and dilution [23]. In this framework, colorimetry appears to be a fundamental analytical methodology to detect and quantify such ingredients selectively by color variation with high simplicity, low cost, and often reduced times for sample preparation and analysis [18,19]. Recently, we have reported the selective detection and quantification of L-DOPA in the copresence of carbidopa [24] or, vice versa, the selective detection and quantification of carbidopa in the same formulations [25] for two commercial brand and generic drug tablets for the treatment of Parkinson’s disease. In the first case, we took advantage of the spontaneous oxidation and color development of L-DOPA by using the generation, in presence of magnesium acetate and dimethyl sulfoxide, of the purple melanochrome as the colorimetric reporter, whereas the synthetic analog carbidopa appeared uncolored [24]. In the second case, here optimized, we have reported a selective condensation reaction between the hydrazine group from carbidopa and the formyl functional group of the natural flavoring agent vanillin in acidified alcoholic solution, resulting in yellow color development upon the formation of a benzaldazine for carbidopa only via hydrazone intermediate, whereas L-DOPA, lacking the hydrazine group, did not color the solution, as expected by imine formation [25]. That work was based on pioneering works about the reaction between the hydrazine group of carbidopa and *p*-dimethylaminobenzaldehyde, giving an azine as the specific molecular reporter for the quantitative determination of carbidopa in plasma and urine by mass spectrometry and spectrofluorimetry [27,28].

Both the assays developed in our laboratories gave a linear calibration trend for the analytes (L-DOPA or carbidopa) with very good reproducibility (_av_RSD% about 3–4%) and very good sensitivity, with a limit of quantification about 1 mg L^−1^ in any case. The calibration curves for the two tablet formulations were almost superimposable with L-DOPA:carbidopa (4:1 *w*/*w*), as well as the analytes alone (L-DOPA or carbidopa); in standard solution, i.e., the excipients and additives did not interfere with analyte determination [24,25]. Grounded on these results, and with the spectroscopical features of the colored aldazine depending also on the ring substitutions, we decided to explore here the reactivity of other aromatic aldehydes. A simple and inexpensive colorimetric detection and quantification of carbidopa in anti-Parkinson drugs was therefore developed by optimizing the selective condensation reaction between the hydrazine group from carbidopa and the formyl functional group of selected aldehydes in acidified hydroalcoholic solution.

Here, we developed an optical assay by using indole-3-carbaldehyde (I3A) giving a yellow aldazine in EtOH:H_2_O 1:1 (λ_max_~415 nm) at 70 °C for 4 h, and the identity of the products from the aldehyde/carbidopa reaction were confirmed by LC-MS analysis, following the protonated ion of the molecules of interest and the corresponding UV–Vis spectrum, as well as the retention times. A common filter-based plate reader was used for colorimetric data acquisition and carbidopa quantification, providing superior results in terms of the analytical performances (sensitivity, LOD, and LOQ) in comparison with a previous study based on vanillin [25]. The calibration curves in standard solution and drugs were almost superimposable, therefore excluding interference from the excipients and additives. The sensitivity of the I3A-based assay was four times higher, and the limits of detection and quantification were much lower, at least half of the vanillin-based one and, in any case, well below 1 mg L^−1^, with very good reproducibility (_av_RSD% 2–4%) within the linear dynamic range (10 mg L^−1^–50 mg L^−1^, R^2^ ≈ 0.993).

## 2. Materials and Methods

### 2.1. Chemicals and Methods

For the reactions and spectroscopic analyses, the solvents, chemicals, and the following compounds were from Sigma-Aldrich Merck (Milan, Italy): Carbidopa (CD, (2S)-3-(3,4-dihydroxyphenyl)-2-hydrazinyl-2-methylpropanoic acid, CAS 28860-95-9), levodopa (L-DOPA, (2S)-2-amino-3-(3,4-dihydroxyphenyl)propanoic acid, CAS 59-92-7), p-anisaldehyde (4-methoxybenzaldehyde, CAS 123-11-5), vanillin (4-hydroxy-3-methoxybenzaldehyde, CAS 121-33-5), catechaldehyde (3,4-Dihydroxybenzaldehyde, CAS 139-85-5), Terephthalaldehyde (1,4-Benzenedialdehyde, CAS 623-27-8), Amylcinnamaldehyde ((2Z)-2-benzylideneheptanal, CAS 122-40-7), Anthracene-9-carbaldehyde (9-Anthracenecarboxaldehyde, CAS 642-31-9), and indole-3-carbaldehyde (1*H*-Indole-3-carbaldehyde, CAS 487-89-8). Sinemet (200 mg L-DOPA, 50 mg carbidopa, 35 mg excipients) was obtained from MSD (Rome, Italy). Hexal (200 mg L-DOPA, 50 mg carbidopa, 194 mg excipients) was produced by Sandoz (Basel, Switzerland). All chemicals were of analytical reagent grade and used as received without any further purification. MS grade water, acetonitrile, and formic acid were used for the LC-MS analysis and were from Sigma-Aldrich Merck. All solutions were prepared using water obtained from the Milli-Q Water Purification System (resistivity ≥ 18 MΩ cm) (Merk, Millipore, Germany). Stock solutions of reagents were obtained at room temperature by dissolving 99.8 mM I3A in ethanol and 125 g L^−1^ of L-DOPA (633.9 mM) and carbidopa (552.5 mM) in aqueous HCl 1 M. Stock solutions of a brand drug (Sinemet) and the generic drug (Hexal) were prepared by stirring for 10 min one tablet (200 mg L-DOPA, 50 mg CD) in 40.0 mL of 1 M HCl. The working solutions were prepared within 24 h of tablet dissolution. The samples were heated by using a thermos mixer to control the time and temperature of the reaction. For the calibration curves, the final sample solutions were in EtOH:H_2_O 1:1, 40 mM HCl, and 10.0 mM I3A. 

### 2.2. Visible Spectroscopy

Absorbance spectra were acquired at 25 °C using the miniature spectrometer FLAME-T-VIS-NIR-ES (350–1000 nm, Ocean Optics, Dunedin, FL, USA). White light (bulb color 2960 K, output 7 W) from a broadband halogen lamp (HL-2000-FHSA, Ocean Optics, Dunedin, FL, USA) was coupled to the squared cuvette holder through a 200 μm diameter optical fiber. Transmitted light was collected at the opposite side of the holder (cuvette optical path = 1.00 cm) using identical fiber optics directly connected to the spectrometer that was calibrated manually for white/dark before each measurement (scans to average 10, integration time 100 ms, optical resolution 1.33 nm FWHM). The samples were analyzed in quartz cuvettes with an optical path length of 1.0 cm. All colorimetric data from the carbidopa and tablet reactions with aldehydes were acquired in disposable polystyrene 96-well plates (Sarstedt, Milan, Italy) by using an iMark™ microplate visible absorbance reader with optical filters (Bio-Rad, Milan, Italy). Data were fitted using a linear equation:

A_415nm_ = m × [CD] + a, (1)

where A_415nm_ represents the absorbance at 415 nm due to the formation of the aldazine, m represents the slope of the calibration curve, [CD] represents the carbidopa concentration, and a represents the intercept. The limit of detection (LOD) and the limit of quantification (LOQ) were calculated based on the standard deviation (SD) of the mean of the blank values as 3 × SD/m and 10 × SD/m, respectively. The assay reproducibility was reported as the (mean) relative standard deviation % (_av_RSD%) based on 8 replicates for each carbidopa concentration.

### 2.3. LC–MS

The analyses were carried out by high-performance liquid chromatography-ultraviolet detector-electrospray mass spectrometry (HPLC-UV-ESI MS) using a Waters instrument (Waters Italy, Sesto S. Giovanni, Milan, Italy). The apparatus consisted of an Alliance 2695 HPLC with an autosampler, a column oven, and a Diode Array Detector 2996 coupled in series to a Quattro micro triple-quadrupole mass spectrometer equipped with a Z-spray ESI interface. The LC column was a Gemini C18, 100 × 2 mm, 5 µm (Phenomenex, Torrance, CA, USA) The samples were eluted (starting 1 min after injection) with a linear gradient of eluent B (0.1% formic acid a in acetonitrile) in A (0.1% formic acid and 4 mM ammonium acetate in water, pH 4) from 0% to 98% in 14 min, remaining at 98% B for 7 min. The column was re-equilibrated at the initial conditions for 10 min. The flow rate was set to 0.3 mL min^−1^, and the column was thermostated at 35 °C. The injection volume was 20 µL, and no splitter was used between the UV and MS detectors. UV spectra were recorded in the range of 250–600 nm at 2.4 nm resolution and a 0.5 spectra s^−1^ rate. The ESI interface and MS parameters were optimized during the infusion of the analyte solutions. Specifically, they were spray capillary 3.0 kV, cone voltage 22 V, extractor lens 3 V, source temperature 120 °C, and desolvation gas temperature 370 °C. High-purity nitrogen (N_2_) was used as the cone gas and desolvation gas at 20 L h^−1^ and 380 L h^−1^, respectively. The mass spectrometer was operated in the positive ion mode, and data were acquired during a full scan in the *m*/*z* range from 100 to 500 at a 0.5 s scan time.

## 3. Results and Discussion

### 3.1. Aldehyde Selection for Aldazine Formation upon Reaction with Carbidopa

The condensation reaction between the hydrazine or hydrazide group and an aromatic aldehyde leads to the development of a colored azine via a hydrazone intermediate [25,27]. Recently, we have applied this chemical principle to carbidopa quantification in the ethanol/water solution of tablet formulations for the treatment of Parkinson’s disease (Figure 1) [25]. In particular, the reaction of an excess of vanillin (4-hydroxy-3-methoxybenzaldehyde) with the hydrazide group of carbidopa at an acidic pH generated the yellow 4-hydroxy-3-methoxybenzaldazine [25]. 

**Figure 2:**
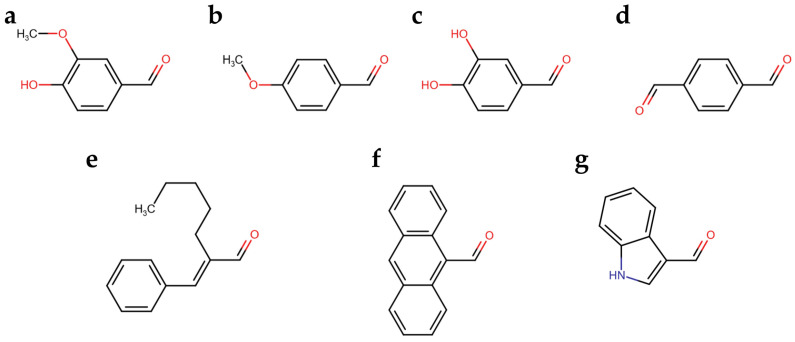
(**a**) Vanillin; (**b**) p-Anisaldehyde; (**c**) Catechaldehyde; (**d**) Terephthalaldehyde; (**e**) Amylcinnamaldehyde; (**f**) Anthracene-9-carbaldehyde; (**g**) Indole-3-carbaldehyde (I3A).

We developed a colorimetric assay considering that the spectroscopical features of the colored aldazine depends on the ring substitutions and on the solvent polarity and pH. Therefore, we explored the reactivity of seven aromatic aldehydes with the hydrazide group of carbidopa, in addition to vanillin, which was previously reported. The reaction was let to progress to the color development of the corresponding azines (Figure 2). In particular, the aromatic aldehydes varied in functional groups (Figure 2b–d) and in double-bond conjugation extensions (Figure 2e–g). 

**Figure 3:**
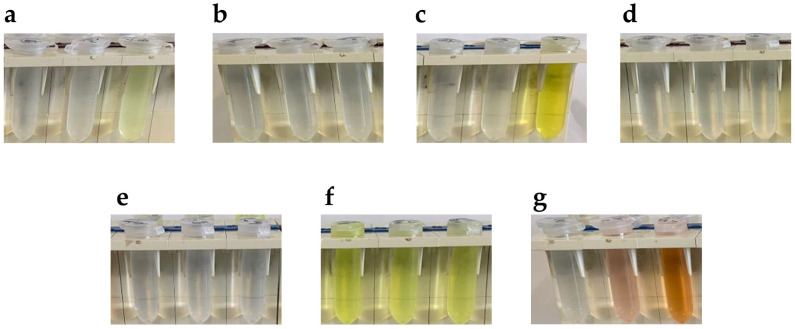
Pictures of 10 mM aldehyde solutions alone (left tube) or in presence of 1 mM carbidopa after 15 min of reaction in ethanol at 20 °C (middle tube) and 70 °C (right tube) for (**a**) Vanillin, (**b**) p-Anisaldehyde, (**c**) Catechaldehyde, (**d**) Terephthalaldehyde, (**e**) Amylcinnamaldehyde, (**f**) Anthracene-9-carbaldehyde, and (**g**) Indole-3-carbaldehyde (I3A).

The first step was to look for aldehydes leaving azine significantly colored, allowing to perform a carbidopa quantitative analysis. In Figure 3 are reported the picture of 10 mM aldehyde solutions alone (left tube) or in presence of 1 mM carbidopa after 15 min of reaction in ethanol at 20 °C (middle tube) and 70 °C (right tube). In this preliminary analysis, as expected by the double-bond conjugation, the only aldehyde giving a colored solution prior to the reaction was anthracene-9-carbaldehyde [29] (Figure 3f). However, with the addition of carbidopa, i.e., the analyte, and the heating at 70 °C, no color change was observed for either anthracene-9-carbaldehyde or with *p*-anisaldehyde (Figure 3b), terephthalaldehyde (Figure 3d), and amylcinnamaldehyde (Figure 3e). All these solutions stayed apparently uncolored, suggesting a minor, if any, azine formation. Conversely, the solutions of vanillin (Figure 3a), catechaldehyde (Figure 3c), and indole-3-carbaldehyde (I3A, Figure 3g) were colored from pale yellow to orange after heating at 70 °C, likely due to azine formation, as already demonstrated for a vanillin reaction with carbidopa [25]. 

### 3.2. Proposed Mechanism of Reaction of Selected Aldehydes and Carbidopa

**Figure 4:**
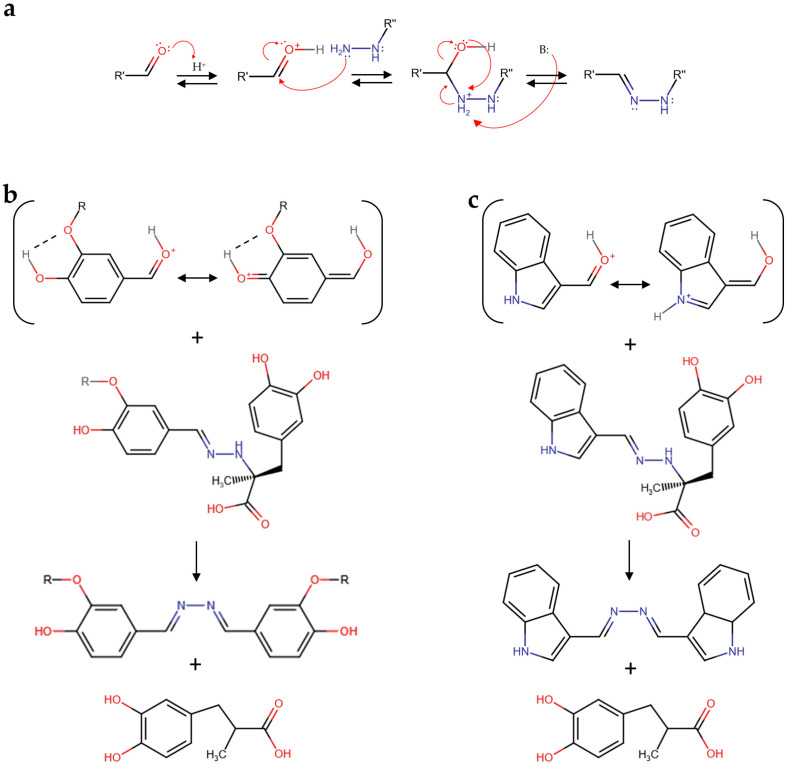
(**a**) Schematic mechanism of hydrazone formation by a reaction between an aldehyde and carbidopa. (**b**) Vanillin (R = CH_3_), catechaldehyde (R = H), and (**c**) indole-3-carbaldehyde (I3A) schematic mechanisms of azine and 3-(3,4-dihydroxyphenyl)-2-methylpropanoic acid formation by reaction between the protonated forms of these aldehydes and the corresponding carbidopa-derived hydrazone.

Although the elucidation of the molecular mechanism of aldehyde reactivity with carbidopa in acidified hydroalcoholic solution is out of the scope of this paper, it is worth noting that the carbidopa hydrazide functional group likely produced a hydrazone with all the aldehydes reported in Figure 3 via nucleophilic attack of the primary nitrogen atom of carbidopa against the protonated formyl group of each aldehyde and subsequent water elimination, as previously reported for vanillin (Figure 4a) [25]. The subsequent reaction of each hydrazone with another molecule of aldehyde to form the correspondent azine [25] appears limited by the steric hindrance. Accordingly, this nucleophilic attack of hydrazone could require a larger stability for the protonated formyl group of the free aldehyde. Being the formyl protonation favored by the resonance effects, i.e., the charge delocalization around the aromatic structure of the aldehydes here described, it is possible that such superior stability, in comparison to the other aldehydes, occurred via electron donation by the oxygen atom in position 4 to the benzaldehyde ring of vanillin and catechaldehyde, favored by the hydrogen bonding between the *ortho*-substituents (Figure 4b). Similarly, the lone pair donation from the nitrogen atom to the pyrrole ring of indole-3-carbaldehyde (I3A) delocalizes the formyl charge by resonance (Figure 4c). Together with the azine, a molecule of 3-(3,4-dihydroxyphenyl)-2-methylpropanoic acid should be formed, likely through a carbocation intermediate that should lead to the loss of chirality, previously identified as a metabolite of carbidopa [27,30].

### 3.3. Visible Spectroscopy of Carbidopa/Aldehyde Reaction Products

**Figure 5:**
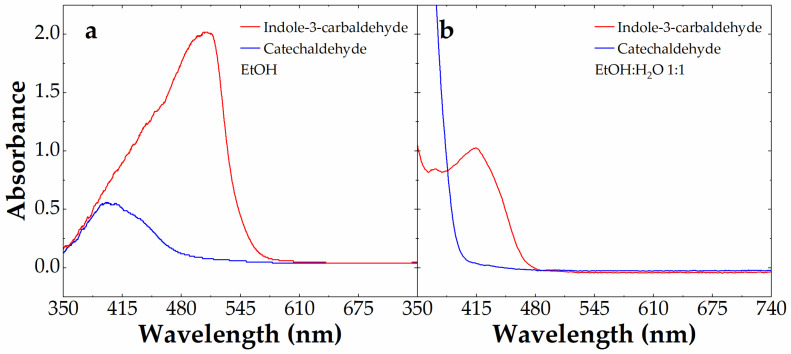
Absorbance spectrum of 10 mM catechaldehyde (blue line) and indole-3-carbaldehyde (red line) alone or in the presence of 0.88 mM carbidopa after 4 h at 70 °C in EtOH (**a**) and EtOH:H_2_O 1:1 (**b**).

We focused the analysis only on aldehydes giving new colored azines, namely catechaldehyde (blue line) and indole-3-carbaldehyde (red line). Figure 5 shows the visible absorbance spectra of these aldehydes at 10 mM in the presence of 0.88 mM carbidopa after 4 h at 70 °C in EtOH (a) and EtOH:H_2_O (1:1) (b). These conditions were chosen to maximize the color formation observed in Figure 3. With the visible absorbance of azine derived by I3A being larger than the one derived by catechaldehyde above 400 nm, we decided to exploit the colorimetric features of I3A to build a calibration curve after the LC-MS characterization (see below). In particular, we focused on the EtOH:H_2_O (1:1) mixture, because, although associated with a lower azine absorbance and λ_max_, this would be the typical solvent conditions obtained by mixing 1:1 (*v*/*v*) the I3A reactant in EtOH with water-based samples, from drugs to biological matrices.

### 3.4. LC–MS Analysis of Carbidopa/I3A Reaction Products

**Figure 6:**
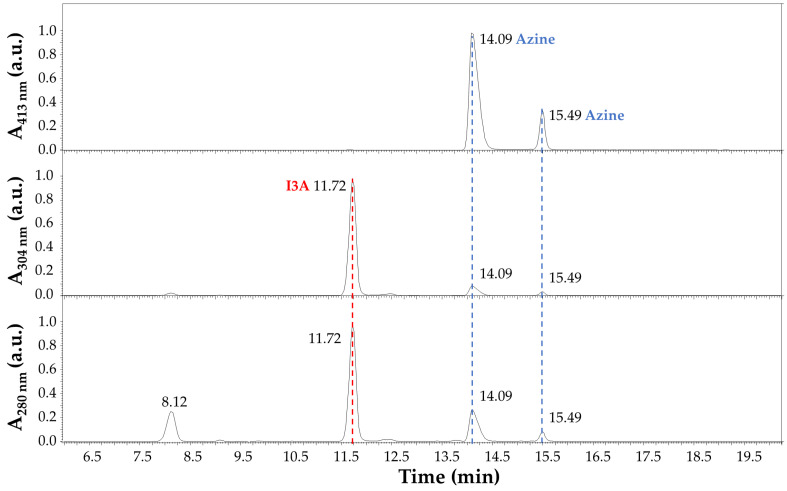
Chromatographic profile of carbidopa/I3A reaction products after 4 h at 70 °C in EtOH:H_2_O (1:1) with 40 mM HCl. Absorbance (a.u.) at 280 nm (**lower** panel), 304 nm (**middle** panel), and 413 nm (**upper** panel) was recorded by using the Diode Array Detector (DAD). The peak corresponding to I3A (C_9_H_7_NO) is highlighted by a red dashed line, and its absorbance is limited to the UV region (280 nm and 304 nm). Peaks corresponding to a I3A azine molecule (C_18_H_14_N_4_) are highlighted by blue dashed lines, and their absorbance covers UV and visible regions (280 nm, 304 nm, and 413 nm).

**Figure 7:**
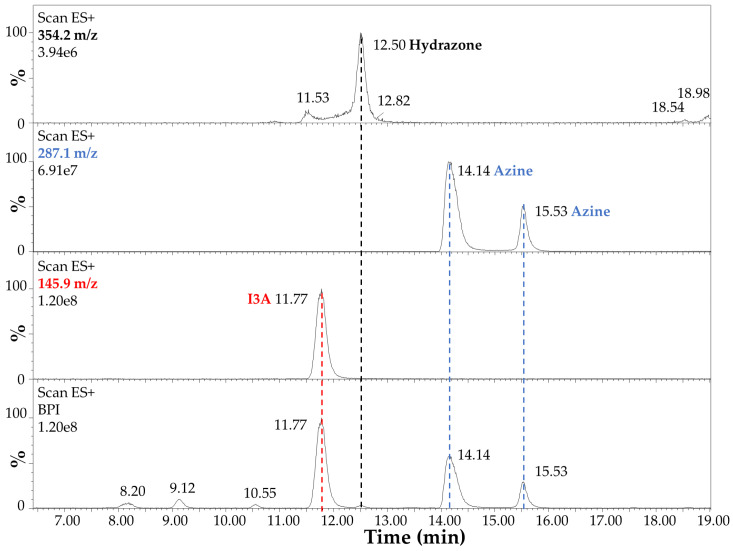
Electrospray mass spectrometry analysis in positive ion mode (Scan ESI^+^) of carbidopa/I3A reaction products after 4 h at 70 °C in EtOH:H_2_O (1:1) with 40 mM HCl. The highest intensity signals (base peak intensity, BPI) are reported in the lower panel. The [M + H]^+^ ion corresponding to I3A (C_9_H_7_NO, 145.9 *m*/*z*) is highlighted by a red dashed line. The [M + H]^+^ ions corresponding to I3A azine molecule isomers (C1_8_H_14_N_4_, 287.1 *m*/*z*) are highlighted by blue dashed lines. The [M + H]^+^ ion corresponding to I3A-carbidopa hydrazone (C_19_H_19_N_3_O_4_, 354.2 *m*/*z*) is highlighted by a black dashed line.

The LC-MS analysis of the reaction products after 4 h at 70 °C in EtOH:H_2_O (1:1) and 40 mM HCl is summarized in Figure 6 and Figure 7. In detail, the chromatographic profile recorded by using the Diode Array Detector (DAD) at 280 nm (Figure 6) shows four main peaks in the lower panel in our experimental conditions (see Section 2.3 LC-MS). The first one at 8.12 min was unidentified but tentatively associated with small impurities and an instrumental background. This peak absorbed at 280 nm only. The second peak in Figure 6 at 11.72 min (red dashed line) was ascribed to I3A (C_9_H_7_NO), with absorbance at 280 nm and 304 nm, as expected by the indole ring, and identified by mass spectrometry analysis, assigning a 145.9 *m*/*z* value at 11.77 min in Figure 7, corresponding to the [M + H]^+^ ion. This signal was also confirmed by comparison with a chemical standard of I3A analyzed in the same conditions. The third and fourth peaks in Figure 6 at 14.09 and 15.49 min (blue dashed lines) were associated with a I3A azine molecule (C_18_H_14_N_4_). Both peaks showed large absorbance at 413 nm (yellow), whereas there was no significant absorbance at this wavelength by the other peaks, as expected. The mass spectrometry confirmed, with a value of 287.1 *m*/*z*, the identity of these [M + H]^+^ ions, with retention times of 14.14 and 15.53 min, respectively (Figure 7). The remarkable splitting and difference in elution time for these peaks is here attributed to azine stereoisomers generated by a different mechanism of synthesis with respect to previously reported I3A-hydrazine condensation [31]. These isomers are likely associated with different double-bond configurations of the azine region and/or with different conformations for the rotation around the single bond between the nitrogen atoms limited by steric hindrance, analogous to allylic strain [32], to which the indole rings derived from I3A molecules contribute, in large part. However, further characterization would be necessary for a full description. Finally, a peak attributed to I3A-carbidopa hydrazone (C_19_H_19_N_3_O_4_), with a *m*/*z* value of 354.2 for the [M + H]^+^ ion, was found in trace amounts at about 12.50 min in Figure 7 (black dashed line), supporting the efficiency of I3A-to-azine conversion. A carbidopa molecule was not found (226.2 *m*/*z*) in our chromatographic conditions, likely requiring an LC system with eluents for ion pairing, which is not compatible with the other components here analyzed.

### 3.5. Colorimetric Quantification of Carbidopa in Tablets by Using of I3A Azine as a Molecular Probe

**Figure 8:**
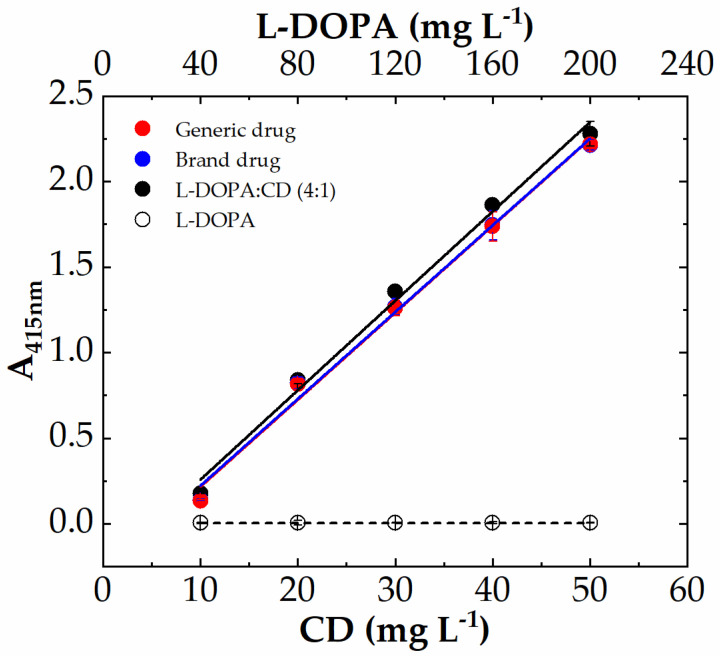
Colorimetric calibration curves of carbidopa (CD) in drug formulation L-DOPA:CD (4:1 *w*/*w*, black circles), Sinemet (blue circles), and Hexal (red circles), followed by the means of aldazine formation (λ_max_ 415 nm) upon reaction with 10 mM I3A for 4 h at 70 °C in EtOH:H_2_O 1:1 and 40 mM HCl. The carbidopa content increased from 10 mg L^−1^ to 50 mg L^−1^, whereas L-DOPA increased from 40 to 200 mg L^−1^, also without carbidopa (white circles). Each point represents the mean ± SD of 8 replicates on a 96-well microplate. Data were fitted according to linear Equation (1).

**Figure 9:**
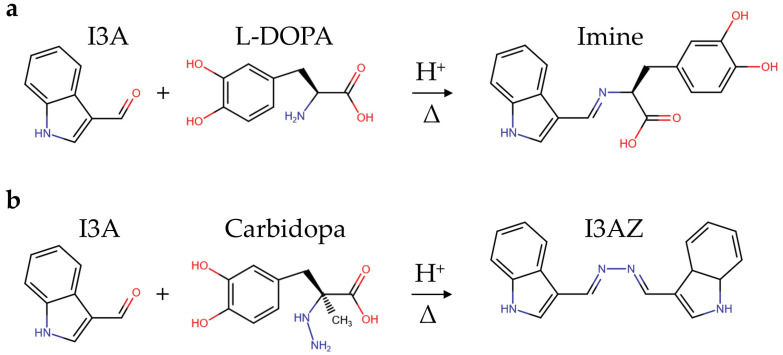
Scheme of the condensation reaction between the formyl functional group of indole-3-carbaldehyde (I3A) in acidified alcoholic solution and (**a**) the hydrazine group from carbidopa, giving the yellow indole-3-carbaldehyde azine (I3AZ), or (**b**) the amine group from L-DOPA, giving the uncolored imine.

The experimental conditions here used for I3A azine (I3AZ) synthesis and LC-DAD-MS characterization, namely 10.0 mM I3A in EtOH/H_2_O 1:1 (*v*/*v*) in the presence of 40 mM HCl at 70 °C for 4 h, were applied for the selective detection of carbidopa (CD) in the copresence of L-DOPA and also in pharmaceutical formulations for the treatment of parkinsonism, following the yellow color development (λ_max_ 415 nm) upon the reaction of carbidopa with the aldehyde. Figure 8 shows the four calibration curves for L-DOPA (white circles), the binary mixture of pure components L-DOPA:CD (4:1 *w*/*w*) (black circles), the brand drug (blue circles), and the generic drug (red circles). The curve associated with L-DOPA standard solutions is flat over the entire concentration range here explored (40–200 mg L^−1^), as expected, because the imine molecule formed by the reaction with I3A (Figure 9a) does not show any significant visible absorbance. On the contrary, all the solutions containing carbidopa were colored yellow upon I3AZ molecule formation (Figure 9b), either the real commercial formulations, containing 200 mg of levodopa, 50 mg of carbidopa, and numerous excipients per tablet (see Materials and Methods) or their corresponding simplified formulation containing the L-DOPA:CD (4:1 *w*/*w*) mixture only. 

The absorbance values at 415 nm were collected for each carbidopa concentration (eight replicas) by using a filter-based plate reader and disposable 96-well microplates. The data were linearly fitted according to Equation (1) (R^2^ ≈ 0.993) after blank subtraction, and the resulting fitted figures of merit are reported in Table 1. The carbidopa-containing samples show almost superimposable curves (Figure 8) that are visually indistinguishable for the drugs, as numerically highlighted by the very close m-values, corresponding to the slope of the I3AZ absorbance recorded at 415 nm. These results underlie the absence of any matrix effect, eventually attributable to these drugs’ different formulations, as well as the absence of interference from of L-DOPA, present at a high content. Moreover, the assay shows very good reproducibility (_av_RSD% 2–4) within the linear dynamic range here explored, and the limit of quantification resulted well below 1 mg L^−1^ in any case, namely 0.379 ± 0.018 mg L^−1^, 0.329 ± 0.016 mg L^−1^, and 0.296 ± 0.009 mg L^−1^ for L-DOPA:CD (4:1 *w*/*w*), the brand drug, and the generic drug, respectively. Finally, all these analytical performances and, in particular, the much higher sensitivity displayed by the assay, resulted in significant improvement of our previously reported work, using a vanillin reaction with the hydrazide group of carbidopa (Table 1) [25], reported here for comparison. This boosted finding was achieved thanks to the rational design of indole-based azine with a more extended double-bond conjugation.

## 4. Conclusions

We developed a colorimetric assay for the quantification of carbidopa in medications for the treatment of Parkinson’s disease, following the pioneering works about the reaction between hydrazine group of carbidopa and *p*-dimethylaminobenzaldehyde, giving an azine as the specific molecular reporter for the quantitative determination of carbidopa by mass spectrometry and spectrofluorimetry in plasma and urine [27,28]. Analogously, the method presented here was based on the aldazine formation and yellow color development upon selective condensation reaction between the analyte carbidopa and the reactant indole-3-cabaldehyde, irrespective of the L-DOPA and excipient contents (Figure 9). The reactant was chosen among seven aromatic aldehydes to obtain an aldazine with an extended double-bond conjugation. The data were acquired by using a common filter-based microplate reader and disposable 96-well microplates, emphasizing the affordability of the method for the quality control of pharmaceutical formulations. The calibration curves for the brand and generic drugs in tablets were superimposable to the mixture of the L-DOPA:CD standards (4:1 *w*/*w*) within the linear dynamic range (10–50 mg L^−1^) with excellent reproducibility (_av_RSD 2–4%). The figures of merit of this assay and, in particular, the shown sensitivity are superior to our previous work based on a vanillin reaction with carbidopa. The limit of quantification (LOQ), well below 1 mg L^−1^, could favor future applications of such methodology to the analysis of urine samples from patients upon drug therapy for PD, representing noninvasive carbidopa monitoring; moreover, the proposed approach could be applied, using I3A, for carbidopa derivatization in biological matrices for the improvement of the sensitivity of the several carbidopa quantification methods by means of LC-MS [16,33,34,35], capillary zone electrophoresis with UV detection [17], chemometrics-assisted spectrophotometric methods [20,21,22], voltammetry [23], and NMR [36].

## Figures and Tables

**Table 1:** Comparison of the colorimetric quantification of carbidopa (CD) in tablets by means of aldazine formation in the presence of vanillin [25] or I3A (this work).

^1^ Sample	Aldehyde	^2^ m (L g^−1^)	R^2^	^3^ LOD (mg L^−1^)	^4^ LOQ (mg L^−1^)	^5^ _av_RSD (%)	Ref.
L-DOPA:CD	I3A	52.3 ± 2.5	0.993	0.114 ± 0.006	0.379 ± 0.018	2.1	This work
	Vanillin	13.2 ± 0.3	0.987	0.184 ± 0.005	0.615 ± 0.015	3.5	[25]
							
Brand drug	I3A	50.7 ± 2.5	0.993	0.099 ± 0.005	0.329 ± 0.016	3.7	This work
	Vanillin	13.4 ± 0.2	0.996	0.215 ± 0.003	0.778 ± 0.010	3.7	[25]
							
Generic drug	I3A	50.8 ± 2.4	0.994	0.089 ± 0.003	0.296 ± 0.009	3.5	This work
	Vanillin	13.2 ± 0.2	0.995	0.342 ± 0.006	1.140 ± 0.019	3.4	[25]

^1^ All samples include L-DOPA:CD (4:1 *w*/*w*), but only the drugs include the excipients; ^2^ m, slope of the calibration curve in Equation (1) ± SD; ^3^ LOD, limit of detection ± SD; ^4^ LOQ, limit of quantification ± SD; ^5^ _av_RSD(%), mean standard deviation calculated between 10 and 50 mg L^−1^ (8 replicates).

## Data Availability

The data will be made available upon request.
